# Randomized Controlled Trial of the Effectiveness of Genetic Counseling and a Distance, Computer-Based, Lifestyle Intervention Program for Adult Offspring of Patients with Type 2 Diabetes: Background, Study Protocol, and Baseline Patient Characteristics

**DOI:** 10.1155/2012/831735

**Published:** 2012-04-26

**Authors:** M. Nishigaki, Y. Tokunaga-Nakawatase, J. Nishida, C. Taru, I. Miyawaki, H. Sanada, K. Kazuma

**Affiliations:** ^1^Department of Adult Nursing, School of Health Sciences and Nursing, Graduate School of Medicine, The University of Tokyo, 113-0033 Tokyo, Japan; ^2^Department of Health Care Center, Social Insurance Central General Hospital, 169-0073 Tokyo, Japan; ^3^Division of Development Sciences for Practical Nursing, Department of Nursing, Faculty of Health Sciences, Graduate School of Health Sciences, Kobe University, 654-0142 Hyogo, Japan; ^4^Department of Gerontology Nursing, School of Health Sciences and Nursing, Graduate School of Medicine, The University of Tokyo, 113-0033 Tokyo, Japan

## Abstract

Relatives of type 2 diabetic patients are at a high risk of developing type 2 diabetes and should be regarded as target of intervention for diabetes prevention. However, it is usually hard to motivate them to implement preventive lifestyle changes, because of lack of opportunity to take advises from medical professionals, inadequate risk perception, and low priority for preventive behavior. Prevention strategy for them therefore should be highly acceptable and suited for them. The parallel, three-group trial is now being conducted to investigate the effects of genetic counseling and/or a computerized behavioral program on the prevention of type 2 diabetes in that population. The preventive strategies used in this study could provide a novel solution to the numbers of genetically high-risk individuals, if found to be effective. The objective of this paper is to describe the background, protocol, and baseline patient characteristics of the trial.

## 1. Background

The number of patients with type 2 diabetes has rapidly and continuously increased in recent years and has become a global burden. Development and implementation of strategies to prevent type 2 diabetes are therefore urgently required. Identification of a high-risk population is one of the most important steps in the development of effective and efficient prevention strategies. Several risk factors for type 2 diabetes have been identified in epidemiological studies. Obesity is the most common risk factor for type 2 diabetes, and a vast amount of literature has reported on interventions aiming to reduce obesity.

 The effectiveness of lifestyle intervention for diabetes prevention has been confirmed by large, randomized, controlled trials in both Western countries [1–3] and Asian countries [4–7]. The subjects of these studies were at high risk for developing diabetes, being obese, and/or having impaired glucose tolerance. The goal of lifestyle interventions in these studies was mainly weight reduction. All of these studies definitely succeeded in preventing diabetes, while weight reduction patterns differed between Asian and Western countries. Studies in Asian countries showed a preventive effect with a smaller weight reduction (0 to 2.5 kg/year) [6, 7] than those in Western countries (4.2 to 7.0 kg/year) [2, 3]. These results reflect obvious differences in baseline body mass index (BMI) among different regions. The target population in preventive research trials usually has a BMI of ≥30 kg/m^2^ in Western countries and about 25 kg/m^2^ in Asian countries [8]. Recent global epidemiological meta-analysis has shown that BMI is strikingly lower in Asian populations compared with Oceania, Australasia, Europe, and North America [9]. Among developed countries, Japanese males have the lowest mean male BMI (23.5 kg/m^2^) and Japanese females have nearly the lowest mean female BMI (21.4 kg/m^2^) [9]. Moreover, Japanese patients with diabetes characteristically have a relatively low BMI [10]. Focusing on obesity would therefore be a less efficient method of identifying high-risk individuals in Asian countries than in Western countries, and it is necessary to focus on risk factors other than obesity.

Etiologically, type 2 diabetes results from a complicated combination of genetic and environmental factors. Individuals genetically predisposed to type 2 diabetes therefore represent an important target for preventive strategies. Some studies have suggested that the Japanese are genetically predisposed to developing diabetes because they have a higher frequency of some diabetes susceptibility genes than Caucasians [11–13]. A recent study reported a diabetes susceptibility gene specific to lean (BMI <24 kg/m^2^) Japanese people [14]. These findings suggest that genetic considerations are especially important in diabetes prevention strategies for the Japanese.

Family history is a well-known risk factor for type 2 diabetes and has been used to screen high-risk populations [15–17] from both genetic and environmental viewpoint. Offspring of patients with type 2 diabetes have a higher risk of developing the disease since they are likely to share the same genetic predispositions and have similar lifestyle habits as their parents [18]. Individuals with an affected first-degree relative have a 2.3- to 5.5-fold higher risk of developing type 2 diabetes, independent of sex, age, race/ethnicity, BMI, and other demographic characteristics [19]. Family history is thus a useful tool for detecting genetically high-risk populations in this postgenomic era [20, 21].

 Some studies have already revealed positive effects of lifestyle interventions for dietary habits in relatives of diabetic patients [22–24], and of increasing recognition of diabetes risk [25]. These results suggest that intervention for relatives would be effective if delivered in a useful way. However, it is difficult for medical professionals to reach the relatives of patients in both research and clinical settings, because the relatives rarely visit hospitals unless they get ill. One way to contact relatives to offer general genetic counseling and promote preventive behavior is to utilize patient as intermediaries between medical professionals and relatives [26, 27]. Unfortunately, using patients in this role had not been demonstrated to be effective in diabetes. The rates of participation in lifestyle intervention programs offered to relatives through patients have been reported to be disappointingly low (5–13.5%) [24, 28], and advice about lifestyle modification given by patients was reported to have no effect on their offspring's preventive behavior [29].

These results suggest that it is difficult to motivate relatives to implement preventive lifestyle changes, especially through patients. It is therefore essential for diabetes preventive strategies to establish a way to contact relatives directly, in order to provide lifestyle intervention programs and maintain compliance in such programs.

One of the most common opportunities for medical professionals to make contact with the offspring of patients with type 2 diabetes is at the time of a medical checkup. In Japan, employers have a legal obligation to allow their employees an annual health checkup. Additionally, the Japanese Ministry of Health, Labour and Welfare enacted the Specific Health Checkup (SHC) in 2008 that aims to identify individuals at high risk for the development of metabolic syndrome in both employed and nonemployed populations. The SHC is mainly undertaken by municipal governments, who will be penalized by central government if they cannot achieve the target consultation rate (e.g., 65% by 2012). Most of the Japanese population is therefore expected to undergo medical checkups. Subjects are usually asked during the checkup if they have a family history of common diseases such as stroke, cardiovascular disease, infectious disease, lipidemia, or diabetes. Unfortunately, this information about family history has not been fully utilized as a screening tool for implementation of intervention to prevent type 2 diabetes [30, 31]. The current study therefore aims to screen for high-risk individuals using information about family history, and then contact subjects directly to provide preventive intervention.

As mentioned above, it is difficult to motivate the relatives of patients with type 2 diabetes to implement preventive lifestyle changes. For high-risk individuals to become actively involved in prevention, recognition of the risk of acquiring the disease is crucial [32, 33]. Risk education is therefore important as the first step in motivating relatives of patients. Information about disease susceptibility is sometimes a psychological burden for high-risk people, especially those who are genetically predisposed [34]. Genetic risk education should therefore be provided as a component of genetic counseling, which is the medical/psychosocial process that disease predisposed subjects are advised of the consequences, nature, and management of an inherited disorder, even though previous research has shown that such information mainly has a favorable effect on psychological issues [25, 35]. However, genetic counseling often seems to be less feasible in the health checkup setting since it takes more time than usually available at an outpatient clinic. It was therefore essential to develop a counseling tool which enables medical professionals to explain genetic diabetes susceptibility and diabetes prevention quickly and adequately. The authors developed a six-page booklet based on the traditional health belief model [33], which can be utilized for brief genetic counseling including risk education about type 2 diabetes. In the current study, brief genetic counseling by a certified genetic counselor was provided to subjects using the booklet. Then the current study tried to investigate the effect of that genetic counseling on participant's compliance to the lifestyle intervention.

To maintain compliance with lifestyle interventions, the interventions should be easily available. It is particularly difficult to motivate the offspring of type 2 diabetic patients to receive preventive intervention, as they are generally healthy and have other demands on their time. Previous research has indicated that healthy subjects prefer correspondence format programs to face-to-face format programs for the delivery of health education [36]. Correspondence format programs include telephone, internet, CD-ROM, and mail formats. The printed format may be particularly advantageous as the material is more likely to be read, saved, and perceived as personally relevant than information presented on a CD-ROM [37, 38]. Even though telephone programs are more personal and immediate and involve more natural language than mail programs [39], both telephone and mail programs are effective in reducing the health risk status of participants [40]. Mail programs place fewer time constraints on people and are more readily available than internet programs [39]. Mail format, which is easily available, is therefore considered suitable for maintaining compliance with preventive interventions for potentially high-risk relatives.

To provide effective lifestyle interventions, medical professionals should comprehensively assess subjects and deliver individualized information, which can be achieved efficiently using computer-tailored health education [41, 42]. Computer-tailored lifestyle intervention is a promising health education technique, particularly for (printed) nutrition education [43] and is well suited to modification of complex health-related behaviors (e.g., by providing feedback). Previous studies have shown that computer-tailored intervention involving questionnaires and tailored messages can result in behavioral changes and weight loss in healthy people [44–46]. Furthermore, computer-tailored interventions have emerged as a new and cost-effective type of health promotion program because they enable personalization of health education without the high cost of personal counseling [43]. The computerized behavioral program is therefore expected to be an effective and feasible intervention tool.

 The objective of this parallel, three-group trial is to investigate the effects of printed computer-tailored lifestyle intervention on favorable lifestyle change, and the effects of genetic counseling about prevention and hereditary risk of type 2 diabetes on the compliance to the life style intervention.

## 2. Methods

### 2.1. Design

This study is a parallel, three-group, randomized, controlled trial investigating the effects of printed computer-tailored lifestyle intervention (LI) on favorable lifestyle change, and the effects of genetic counseling (GC) about prevention and hereditary risk of type 2 diabetes on the compliance to the life style intervention.

Data collected at preintervention, immediately post-GC (baseline), and at 1 week and 3, 6, and 12 months post-GC will be analyzed. The design, conduct, and reporting of the study adhere to the Consolidated Standards of Reporting Trials (CONSORT) guidelines [47].

### 2.2. Subjects

#### 2.2.1. Eligibility Criteria

Subjects were included if they had a first-degree relative with type 2 diabetes and were aged 30–60 years. Those who were already diagnosed with type 2 diabetes or metabolic syndrome, or were already receiving lifestyle intervention, were excluded.

#### 2.2.2. Setting

Subject recruitment was carried out in the medical checkup department of a general hospital in Tokyo.

### 2.3. Interventions

Subjects were randomized into three groups: genetic counseling and lifestyle intervention (GC&LI), lifestyle intervention (LI), and control.

#### 2.3.1. Genetic Counseling

After written consent was obtained, subjects underwent genetic counseling with a certified genetic counselor. The counseling session used the booklet developed by the authors, which has been described elsewhere [48]. Briefly, the booklet consists of four sections, each reflecting a core element of the health belief model, with the following information provided in each section. (1) *Perceived seriousness.* Information on the symptoms and complications of diabetes. (2) *Perceived susceptibility.* Information on the drastic increase in the number of diabetic patients, and the implications of genetic-environmental interactions. Causes of diabetes, such as genetic predisposition, high-fat foods, and/or a sedentary lifestyle [11, 49]. Information on genetic predisposition, decreased insulin secretion, and the decreased insulin sensitivity caused by a high-fat meal [21]. Advice that individuals with an affected first-degree relative have a 2.3–5.5-fold higher risk of developing type 2 diabetes [19] since such individuals seem to have similar genetic predisposition and lifestyle. (3) *Perceived benefits.* Advice that the risk of acquiring diabetes can be modified by a low-fat diet and increased physical activity (PA) [50, 51]. (4) *Perceived barriers.* A summary of concrete methods to modify diet and PA, and a recommendation to refer to professionals for individualized prevention [50, 51]. The total time for the counseling session was approximately 10 min.

#### 2.3.2. Printed, Computer-Tailored Lifestyle Intervention

Lifestyle intervention in this study features tailored and concrete lifestyle recommendations in a computer-based, non-face-to-face format. The intervention will use Lifestyle Intervention Support Software for Diabetes Prevention (LISS-DP). Contents of LISS-DP are developed based on lifestyle intervention protocol for secondary or tertiary prevention in type 2 diabetes patients, which has been developed by one of authors [52, 53].

The intervention strategy consists of the following processes: (1) lifestyle and background data collection by self-administered questionnaire; (2) generation of tailored recommendations; (3) output of tailored recommendations; (4) delivery of the recommendations via mail.

**(1) Lifestyle and Background Data Collection by Self-Administered Questionnaire**

LISS-DP requires information about the subject's current diet and PA to identify risky or favorable behavior. Current lifestyle behaviors will be assessed using a self-administered questionnaire. The questionnaire is based on two measurement scales: evaluation scale for self-management behavior related to physical activity of type 2 diabetic patients (ES-SMBPA-2D) [54] and Dietary Self-Management Behavior Questionnaire (DSBQ) [55]; which were developed by the authors. These scales measure dietary and PA self-management behaviors in diabetic patients and consist of 32 and 91 items, respectively. We modified these scales to assess preventive dietary and PA behaviors in healthy relatives of patients with type 2 diabetes. Items were chosen for each scale according to the following criteria: dietary behavior shown in previous research to have a significant relationship with total energy intake [52, 53], PA behavior shown to have a significant relationship with energy expenditure [54], and important preventive behaviors as judged by an expert panel. As a result, a lifestyle assessment questionnaire consisting of 53 items was developed, with 15 items assessing “recommended dietary behaviors (RD)” (Table 1), 21 items assessing “recommended physical activity behaviors (RP)” (Table 2), 16 items assessing “nonrecommended dietary behaviors (ND)” (Table 3), and a single item assessing “nonrecommended physical activity behavior (NP)” (Table 3). Questionnaires ask the subjects to indicate the frequency of each behavior on five-point scale which will be converted to a numerical score: never (0), rarely (1), sometimes (2), often (3), or always (4).

**(2) Generation of Tailored Recommendations**

LISS-DP will be used to assess each subject's lifestyle behaviors and generate individualized recommendations according to the following algorithms (Figure 2). First, subjects will be divided into four groups according to their combination of risk factors, sex, and physical activity level: male/high-PA group (A), male/low-PA group (B), female/high-PA group (C), and female/low-PA group (D). A high physical activity level is defined according to the national health promotion policy (Healthy Japan 21) as ≥30 min of moderate physical activity ≥2 days/week. LISS-DP will then select the items to be assessed in each risk group. In addition to these selected items, subjects with obesity-related risk factors, high BMI, and/or large waist circumference will be assessed using items which have a relationship with obesity. Cut-off point for each obesity-related risk factor was set based on those of national screening program (SHC, mentioned previously). If subjects answer “never” or “rarely” to one of the selected RD or RP items, that item will be identified as a behavior which the subject should aim for. If subjects answer “often” or “always” to one of the selected ND, that item will be identified as a behavior which the subject should refrain from. If subjects answer “always” to NP item, it will be also identified as a behavior which the subject should refrain from. Additionally, if subjects answer “often” or “always” to one of the RD or RP items, that item will be identified as a behavior which should be continued.

**(3) Output of Tailored Recommendations**

Dietary and PA behaviors which should be aimed for, refrained from, or continued by the subject will be printed on a lifestyle advice sheet consisting of computer-based lifestyle recommendations and a free-comment section for use by the clinical diabetes educator.

The lifestyle recommendations consist of five sections. Sections I and II provide positive feedback about favorable behaviors to increase self-awareness about preventive behaviors. RD item(s) and RP item(s) identified as behavior(s) which should be continued will be indicated as follows.

**Section I**

Your current behaviors(s) shown below are effective for maintaining a favorable total energy intake. Be confident in continuing these actions.

**Section II**

Your current behavior(s) shown below are effective for maintaining a favorable physical activity level. Be confident in continuing these actions.

Dietary behavior(s) which should be aimed for will be identified in section III as follows.

**Section III**

The behavior(s) shown below are recommended for maintaining a favorable total energy intake. Please incorporate these into your daily life to achieve favorable dietary habits.

Both dietary and PA behavior(s) which the subject should refrain from will be identified in section IV as follows.

**Section IV**

The behavior(s) shown below would cause an excess of calorie intake. Please refrain from these behaviors.

Physical activity behavior(s) which should be aimed for will be identified in section V as follows.

**Section V**

The behavior(s) shown below are recommended for increasing physical activity. Please incorporate these into your daily life to achieve favorable physical activity levels.

Following the lifestyle recommendations, a clinical diabetes educator will write advisory comments. The advisory comments will mention general recommendations for lifestyle change, and integrate the individual recommendations. After the second intervention, changes in lifestyle since the previous data collection will be also be shown in the advisory comments.

**(4) Delivery of Recommendations via Mail**

The lifestyle advice sheet will be sent to subjects via mail.

#### 2.3.3. Control Group

Subjects allocated to the control group will receive conventional routine care during the study period, including disclosure of medical checkup results and general information about diabetes prevention. This conventional routine care will also be provided to the other two groups. The genetic counseling booklet and three lifestyle advice sheets will be sent to subjects in the control group after the end of the study period.

### 2.4. Primary Outcomes

#### 2.4.1. Changes in Total Energy Intake, Fat-Energy Ratio, and Physical Activity

The primary outcomes of this study are changes in total energy intake, fat-energy ratio, and physical activity levels between baseline and the end of the intervention period. These variables will be measured by self-administered questionnaires. The questionnaires include a dietary measurement scale (Modified-Ministry of Health and Welfare-Food Questionnaire (M-MHW-FQ)) and a physical activity scale (International Physical Activity Questionnaire (IPAQ)). The M-MHW-FQ has been shown to be a reliable method of calculating energy intake and fat-energy ratio [56]. The IPAQ has been shown to be a valid method of assessing physical activity across various life activities, including during leisure activities [57–59].

### 2.5. Secondary Outcomes

#### 2.5.1. Lifestyle Behaviors

Lifestyle behaviors will be assessed by self-administered questionnaires. Details of lifestyle behavior assessments have already been discussed in the intervention section.

#### 2.5.2. Changes in Biomedical Characteristics

 Biomedical data collected from hospital records at baseline and at 12 months (age, BMI, waist circumference, and levels of HbA_1C_, fasting blood glucose, triglyceride, LDL cholesterol, and HDL cholesterol) will be used in the analyses.

#### 2.5.3. Change in Risk Perception and Recognition About Diabetes, and Attitude towards Its Prevention

Subjects' perception and recognition about diabetes and attitude towards its prevention will be assessed before GC session (baseline for these variables) and 1 week after the session in order to evaluate the effect of GC on subjective aspect.

### 2.6. Data Collection

Data collected at the time of recruitment and at 1 week and 3, 6, and 12 months after recruitment will be used in this study. Background characteristics will be assessed at the time of recruitment: whether he/she has impaired glucose tolerance, abnormalities of lipid metabolism, hypertension or hyperuricemia; living status; occupational status; educational status. Baseline values for outcome measures related to lifestyle behaviors will be collected at 1 week after recruitment because participants must spent more than 15 minutes to answer the questionnaire for lifestyle assessment, and it is infeasible to order them about completing it during medical checkup. All questionnaires will be sent and returned via mail, except for the questionnaire completed at the time of recruitment. We will phone participants or mail a reminder if the questionnaire has not been returned until deadline, four weeks after each questionnaire had been sent. Biomedical data were collected at recruitment and will be collected again at 12 months.

### 2.7. Sample Size

We had conducted preliminary intervention study for offspring of type 2 diabetes patient [24]. The intervention in preliminary study was conducted in mail-delivered style, similar to current study, while advice sheet was made by a dietician and a medical fitness therapist in handwriting. After three-times intervention during 6 months, −1.0 SD change was observed in primary outcome (total energy intake). Intervention in current study is computer-based, it means less tailored to individual participant than preliminary study. So observed effect in this study is expected to be modest compared to preliminary study. We calculated the necessary sample size to be 64 per study arm to detect a moderate effect size (0.5 SD), with 80% power at the 5% significance level. Assuming a dropout rate of 10% from the results of a previous similar study [37, 46], 72 participants are needed for each arm.

### 2.8. Randomization

Subjects were randomly allocated to one of three equal-sized groups. Random allocation was performed using a table of random numbers. Researchers were blinded to characteristics of the subjects during randomization.

### 2.9. Blinding

Group allocation obviously cannot be blinded to participants in this study. Therefore, this study will be conducted with a single-blinded design; group allocation was blinded to the clinical diabetes educator and researcher who will analysis the data.

### 2.10. Data Management

 Once questionnaire will be sent back to research institute, one of researchers immediately check the validity of responses. If missing value or outlying response was found, researcher would phone participant to correct these incomplete response. To avoid human error in data input, two research assistants independently input all the responses into computer. After checking one dataset against another one and confirming perfect matching, the dataset will be input into LISS algorithm.

### 2.11. Statistical Analysis

Statistical analysis will be carried out on an intention-to-treat basis. We will summarize continuous variables using means and standard deviations, and categorical variables using counts and percentages.

The effects of GC will be investigated by comparing the GC&LI and LI groups. Similarly, the effects of LI will be investigated by comparing the LI and control groups. Primary outcomes will be analyzed by ANCOVA, adjusting for baseline values. Differences between groups in secondary outcomes at each assessment point will be analyzed by the Student's *t*-test for continuous variables or the Mann-Whitney *U* test for ordinal variables.

In all tests, a *P* value of <0.05 by two-tailed test will be considered significant. Analysis will be performed using SAS 9.2 (SAS Institute, Cary, NC, USA) software for windows.

All study protocols have been approved by the institutional review board of the School of Medicine, The University of Tokyo and Social Insurance Chuo General Hospital. Clinical Trial Registration: UMIN-CTR: UMIN00003589, UMIN00003636.

## 3. Results

**Figure 1:**
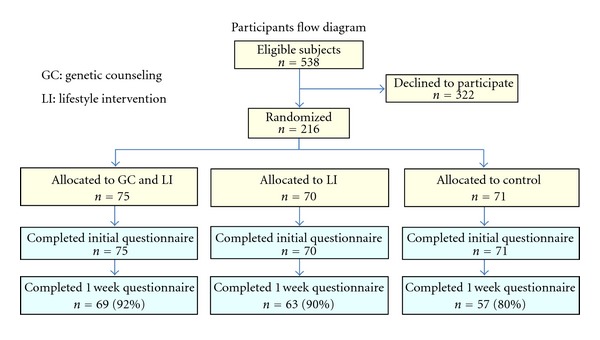


**Figure 2:**
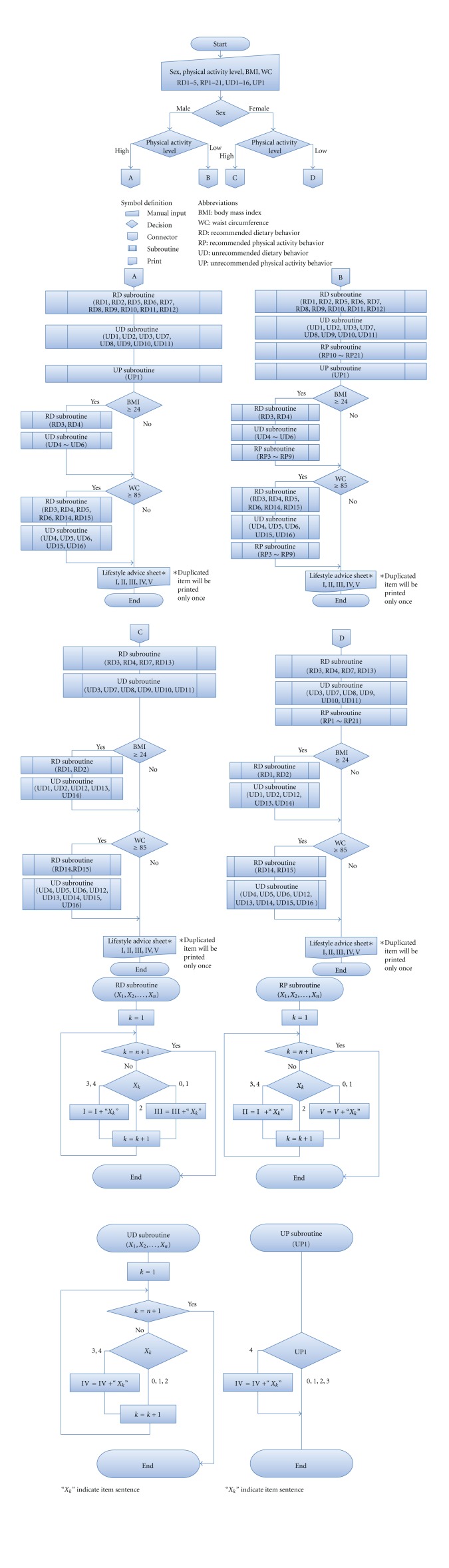
Lifestyle advice sheet output flowchart in LISS-DP.

Figure 1 shows a flow diagram of subject enrollment and initial data collection. During the recruitment period, 538 eligible examinees visited the outpatient department. Of these, 322 (59.8%) declined to participate in the study, leaving 216 subjects who were randomly allocated to one of three groups and completed initial data collection: GC&LI (*n* = 75), LI (*n* = 70), and control (*n* = 71). The baseline questionnaire about lifestyle behaviors was completed by 69 subjects (92%) in the GC&LI group, 63 subjects (90%) in the LI group, and 57 subjects (80%) in the control group.

Table 4 shows the baseline characteristics of subjects. Overall, 61.1% of subjects are male, and the mean age is 45.8 ± 7.8 years. The mean BMI indicates that subjects are in the nonobese range (22.5 ± 3.0 kg/m^2^). All the biomedical characteristics were in the normal range. Mean energy intake was 1777.4 ± 420.8 kcal/day, and fat/energy ratio was 28.9 ± 6.6%. Mean energy expenditure by physical activity was 279.0 ± 510.2 kcal/day.

## 4. Discussion

One of novel challenges in this study is to bring genetic approach into conventional medical checkup system. At present, genetic screening using information about variants of known disease susceptibility genes which has been identified by genome-wide association study is technically available [60]. However, genotype profiling seems to be less useful for individual risk prediction because most of diabetes susceptible genes show not more than 1.4-fold increased risk of type 2 diabetes [61]. Several large-scale epidemiological study have shown that genotype adds only a small amount of additional information to risk prediction models that include common risk factors such as obesity, sex, and family history [62–65]. In addition, people who get genomic profiling and intervention based on their genotype do not modify their lifestyle or attitude towards prevention much, at least in the short term [66, 67]. Therefore, family history is still a useful, clinically available, costless, and etiologically robust diabetes screening tool in this postgenomic era.

The current study presents a preventive strategy targeting individuals with a positive family history of type 2 diabetes but without obesity. This population surely has a risk of developing diabetes, but tends to be overlooked in the current medical checkup system. The interventions undertaken in this study have been developed based on a framework for self-management interventions for secondary or tertiary prevention in patients with type 2 diabetes. Conventional face-to-face lifestyle intervention has been modified to effectively motivate subjects and be easily accepted by them. Genetic counseling including discussion of the hereditary risk of diabetes and of risk management by modification of environmental factors was undertaken to try to motivate subjects. The computer-based and non-face-to-face interventions attempt to address barriers regarding initiation and maintenance of lifestyle modifications in subjects, while also maintaining clinical feasibility.

The agreement rate for participation in the current study was about 40%. Previous intervention studies recruiting adult offspring of patients with type 2 diabetes have reported much lower participation rates (5–13.5%) [24, 28]. Direct recruitment at a medical checkup department is therefore a more effective method for implementation of prevention strategies in individuals with a family history of diabetes than indirect recruitment through patients. However, the acceptance ratio is still not very high, with more than half of eligible subjects refusing to participate. This might cause a sampling bias which could weaken the external validity of the study.

The average health status of study participants is normal, with both BMI and waist circumference in the normal range. Total energy intake at baseline was 29.8 kcal/kg/day, which is already in the favorable target range defined in the Da Qing Study (25–30 kcal/kg/day) [5].

 In conclusion, the current study has achieved a relatively successful recruitment of subjects with a family history of type 2 diabetes but without other common risk factors such as obesity. The interventions designed for this study have specific features which are theoretically advantageous for interventions in subjects who have a genetic predisposition for disease but are currently healthy, and are clinically feasible. The preventive strategies used in this study could therefore provide a novel solution to the numbers of high-risk individuals not participating in preventive programs, if found to be effective.

## Figures and Tables

**Table 1:** Recommended dietary behaviors.

RD1	I use food products with calorie content labels and carefully count calories.
RD2	I try to eat mostly low-calorie foods.
RD3	I eat meat with as little fat as possible.
RD4	I eat boiled meat dishes with reduced fat content (e.g., *shabu-shabu*).
RD5	I chew my food carefully before swallowing.
RD6	I savor my food before swallowing.
RD7	I use low-calorie artificial sweeteners.
RD8	I eat one fish or meat dish with some vegetarian dishes.
RD9	I eat small amounts of a variety of foods.
RD10	I eat a lot of vegetables.
RD11	I make soup with many different kinds of vegetables.
RD12	I eat vegetable dishes including meat and/or fish.
RD13	I try to eat mostly low-calorie foods when dining out (such as vegetables, nonmeat products and nondairy products).
RD14	I select Udon or Soba instead of Chinese noodles in soup.
RD15	I eat vegetable dishes such as a vegetable side dish and/or a vinegar or pickle dish.

**Table 2:** Recommended physical activity behaviors.

RP1	I spend a lot of time shopping.
RP2	I try to increase the number of days I go shopping.
RP3	I take the stairs instead of the elevator.
RP4	I walk or cycle at a brisk pace.
RP5	I stand when taking a train or bus.
RP6	I do not use the TV remote control.
RP7	I stretch or walk during my free time.
RP8	I do the housework myself instead of asking someone else to do it.
RP9	I stretch or walk while watching TV.
RP10	I try to increase the number of days I leave the house.
RP11	I walk or cycle instead of driving.
RP12	I choose a hilly route when walking or cycling.
RP13	I make detours when going somewhere.
RP14	I wear good shoes while walking.
RP15	I choose places with a pleasant environment and interesting sights for walking, jogging, or cycling, to increase my enjoyment of the activity.
RP16	I exercise in all seasons.
RP17	I select a suitable place and time for physical activities.
RP18	I set a daily time for favorite physical activities.
RP19	I make a weekly schedule for favorite physical activities.
RP20	I exercise as leisure activity when on holiday.
RP21	I exercise with family members, friends, or pets.

**Table 3:** Nonrecommended dietary and physical activity behaviors.

ND1	I eat pickles or appetizers (e.g., *tsukudani*) with rice.
ND2	I eat until I feel full.
ND3	I use a lot of sugar when cooking.
ND4	I drink sweet drinks.
ND5	I add sugar to coffee or tea.
ND6	I add milk to coffee or tea.
ND7	I eat broth from noodle dishes.
ND8	I prefer dishes that have too much flavoring or have a strong flavor.
ND9	I use a lot of sauces, salad dressings, salad oil, and so forth.
ND10	I do not decline if I am urged to eat something.
ND11	Once I start to eat, I feel the urge to finish all the food.
ND12	When I feel stressed, I tend to eat without being aware of it, or being conscious of what and how much I eat.
ND13	When I am extremely hungry, I eat food that makes me feel full.
ND14	If it looks appetizing, I eat it.
ND15	I prefer to eat a single-item dish such as curry and rice, pasta, or a bowl of rice with something.
ND16	I prefer to eat fried food.
NP1	I feel a desire to eat more food after exercising.

**Table 4:** Baseline characteristics of study subjects.

	GC&LI		LI		Control	
	*n* = 75		*n* = 70		*n* = 71	
	Mean ± SD or *n* (%)		Mean ± SD or *n* (%)		Mean ± SD or *n* (%)	
Sex: male	40	(53.3)	47	(67.1)	45	(63.4)
Age (years)	46.9	±7.9	44.9	±7.6	45.6	±8.0
Weight (kg)	60	±10.6	62.2	±9.9	63.1	±11.3
Body mass index (kg/m^2^)	22.1	±3.1	22.5	±2.7	22.9	±3.3
Triglyceride level (mg/dL)	98	±56.4	106.3	±73.2	109.4	±72.8
Low-density lipoprotein level (mg/dL)	118.2	±30.6	117.9	±28.0	115.9	±25.7
Fasting blood glucose level (mg/dL)	94.6	±7.5	94.7	±9.3	95.8	±9.4
HbA1C level	5.2	±0.3	5.2	±0.3	5.3	±0.3
Energy intake (kcal/day)^(1)^	1782.8	±415.3	1689.9	±335.5	1867.7	±493.6
Fat/energy ratio^(1)^	29.2	±6.5	29.2	±5.4	28.2	±8.0
Physical activity (kcal/day)^(1)^	228.6	±279.1	243	±296.6	379.8	±816.6
*Medical history*						
Impaired glucose tolerance	0	(0.0)	1	(1.4)	0	(0.0)
Abnormalities of lipid metabolism	10	(13.3)	8	(11.4)	8	(11.3)
Hypertension	6	(8.0)	2	(2.9)	6	(8.5)
Hyperuricemia	4	(5.3)	3	(4.3)	4	(5.6)
*Family history of diabetes*						
Father	50	(66.7)	50	(71.4)	49	(69.0)
Mother	22	(29.3)	17	(24.3)	27	(38.0)
Siblings	8	(10.7)	9	(12.9)	2	(2.8)
*Living with*						
Spouse	51	(68.0)	48	(68.6)	41	(57.7)
Offspring	43	(57.3)	45	(64.3)	33	(46.5)
Father	14	(18.7)	9	(12.9)	13	(18.3)
Mother	18	(24.0)	19	(27.1)	28	(39.4)
*Occupational status*						
Full-time	63	(84.0)	60	(85.7)	62	(87.3)
Part-time	10	(13.3)	8	(11.4)	8	(11.3)
Housekeeping	2	(2.7)	2	(2.9)	1	(1.4)
*Educational status*						
Less than high school	0	(0.0)	1	(1.4)	2	(2.8)
High school	16	(21.3)	18	(25.7)	10	(14.1)
Junior college/technical school	12	(16.0)	12	(17.1)	17	(24.3)
University/college	45	(60.0)	37	(52.9)	38	(54.3)
Graduate school	2	(2.7)	2	(2.9)	4	(5.7)

^(1)^ *n* = 69 in GC&LI, *n* = 63 in LI, *n* = 57 in control.
